# Differential influences of serum vitamin C on blood pressure based on age and sex in normotensive individuals

**DOI:** 10.3389/fnut.2022.986808

**Published:** 2022-11-29

**Authors:** Rui Huang, Linhua Song, Jingbo Zhao, Yuhua Lei, Tian Li

**Affiliations:** Cardiovascular Disease Center, Central Hospital of Enshi Tujia and Miao Autonomous Prefecture, Enshi Clinical College of Wuhan University, Enshi City, Hubei, China

**Keywords:** vitamin C, blood pressure, hypertension, U-shaped relationship, normotensive

## Abstract

**Aim:**

Hypertension is among the most prevalent chronic diseases with diverse etiology, affecting over 1 billion people globally. In numerous studies, vitamin C inversely correlated with blood pressure and was suspected to have antihypertensive properties. Currently, there is conflicting evidence regarding the relationship between vitamin C and blood pressure, with most studies being conducted on hypertensive subjects. The principal objective of this project was to investigate the relationship between vitamin C and blood pressure in normotensive adult subjects.

**Methods:**

A total of 2,533 individuals aged 20 years and above were enrolled in the present study from the National Health and Nutrition Examination Survey (NHANES) 2017-2018. Outcome variables were systolic blood pressure (SBP) and diastolic blood pressure (DBP). Serum vitamin C was regarded as an independent variable. EmpowerStats software and R (version 3.4.3) were used to examine the association between vitamin C and SBP or DBP.

**Results:**

Vitamin C was reversely correlated with both SBP (β = −0.02, 95% CI: −0.03 to −0.00, *p* = 0.0306) and DBP (β = −0.02, 95% CI: −0.04 to −0.01, *p* = <0.0011) after adjusting all covariates. This reverse relationship may be affected by a number of factors, including a person’s gender, age, race, and ethnicity. A U-shaped association between vitamin C and SBP in females and an inverted one between vitamin C and DBP in males were detected, respectively. We further calculated the inflection points at 90.3 μmol/L for females and 40 μmol/L for males. It is somewhat surprising that a reverse U-shaped distribution between vitamin C and SBP and DBP in people over 50 was detected, and the point of inflection of vitamin C were all located at 40 μmol/L.

**Conclusion:**

Vitamin C was negatively correlated with both SBP and DBP in this cross-sectional analysis. However, a U-shaped relationship and an inverted one were also observed in certain people, which implied that, though vitamin C is considered a vital antioxidant, maintaining vitamin C at appropriate levels may be beneficial according to different populations.

## Introduction

Hypertension is among the most prevalent chronic diseases, affecting over 1 billion people globally (1). Concomitantly, the rapidly increasing morbidity of hypertension, which can progress to other cardiovascular diseases and cerebrovascular conditions, severely impairs quality of life around the world (2–4). With advancements in medicine and socioeconomics, recent decades have witnessed a significant improvement in the treatment and control rate of hypertension, especially in developed countries. However, multiple studies are still urgently needed to develop novel and alternative therapeutics and interventions, thus reducing the prevalence of hypertension to a large degree.

The etiology of hypertension is complex and multifactorial, arising from the interplay of lifestyles, physical activities, living environment, and genetic factors. Moreover, numerous population-based epidemiological studies have recognized that multiple dietary factors were associated with hypertension in recent years (5). For instance, extensive evidence has demonstrated that inappropriate consumption of calcium (6, 7), phosphorus (8, 9), sodium (7, 10, 11), potassium (11, 12), magnesium (13, 14) was intricately related with blood pressure. Additionally, various clinical and animal experimental studies have repeatedly revealed that dietary vitamin C and serum vitamin C were associated with blood pressure (15–19). A meta-analysis that included 18 studies found that serum vitamin C was negatively correlated with systolic blood pressure (SBP) and diastolic blood pressure (DBP) (15). Ashor et al. (18) found that vitamin C supplementation can decrease peripheral pulse wave velocity, SBP, and mean arterial pressure in the elderly. Consistent with these conclusions, numerous relevant studies have detected this inverse relationship (16, 17).

The benefit of vitamin C is attributed partly to antioxidative stress response and anti-inflammatory cytokines, thus reducing endothelial cells oxidative damage following arterioles injury in the progression of hypertension (20–22). Therefore, some researchers believe that vitamin C supplementation may contribute significantly to preventing hypertension (18, 23). Nevertheless, some studies have reached a diametrically opposite conclusion (24, 25). A Mendelian randomized study showed that vitamin C supplementation might not aid in preventing any cardiovascular diseases (24). Similar to this study, observational research suggested that although supplementation of fruits and vegetables can increase the concentrations of serum vitamin C and other beneficial substances, it has no anti-oxidative stress effect in 12 weeks (25). In addition, the current research on the relationship between vitamin C and blood pressure was mainly carried out in hypertensive subjects, which may be affected by the use of antihypertensive drugs and the cause of hypertension. Therefore, we use NHANES for the first time to discuss the potential relationship between serum vitamin C and blood pressure in normotensive cases and to develop a promising strategy to prevent hypertension in the pre-clinical stage.

## Materials and methods

### Study population

The dataset in the present study was received from the National Health and Nutrition Examination Survey (NHANES) collected from 2017 to 2018, which contained cross-sectional socio-demographic, dietary, and medical records obtained by questionnaires, standard physical examinations, and laboratory tests conducted in authoritative laboratories. The NHANES database, a population-based survey conducted by the National Center for Health Statistics (NCHS), is a publicly used data set used to record the health status and related personal and lifestyle characteristics of all civilians in the United States. A multi-stage, complex clusters, probabilistic sample design is used for data acquisition and analysis to achieve nationally representative, rather than a simple random sample from the general US population. In particular, the Centers for Disease Control and Prevention (CDC) is responsible for preparing and disseminating data files to provide full access to the data (26–28).

**FIGURE 1:**
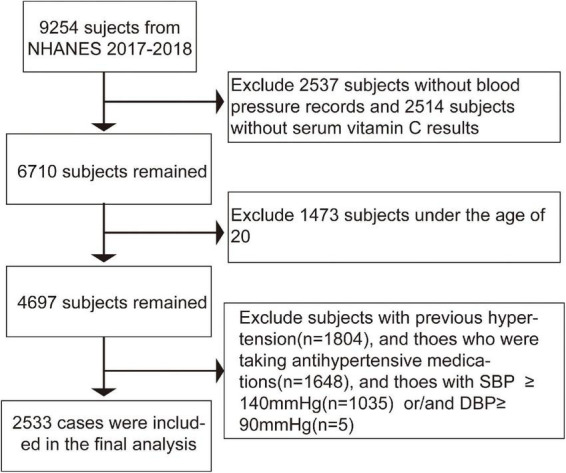
Flow chart of participants selection in the present study.

In the 2017-2018 cycle, there were 9,254 subjects, of which 6,717 cases had valid blood pressure records, and 6,740 individuals were tested for serum vitamin C. We excluded certain participants as follows:(1) individuals with missing serum Vitamin C or blood pressure;(2) those who were diagnosed with hypertension in the past or take antihypertensive medications now;(3) those with SBP ≥ 140 mmHg or/and DBP ≥ 90mmHg. A total of 2,533 subjects were enrolled in the final study. The flow chart of study subjects is shown in Figure 1. NCHS Ethics Review Board supported the research. Furthermore, written informed consent was received from each subject (29).

### Variables

The dependent variable and independent variable of the present study were blood pressure, including SBP and DBP, and serum vitamin C, respectively. Vitamin C was tested and recorded in authoritative laboratories using standard procedures (Details of the test method can be found in Supplementary material 1).

For blood pressure measurement: After 5 min of resting quietly in the seat, once the participant’s maximum inflation level (MIL) is determined, three consecutive blood pressure readings will be obtained. If the blood pressure measurement is interrupted or incomplete, a fourth attempt would be made. All blood pressure measurements were conducted in the Mobile Inspection Center (MEC). The absolute blood pressure is the average of three valid measurements.

Besides, the following variables were included in the present study: age, race/ethnicity, sex, marital status, education level, ratio of family income to poverty, alcohol consumption, smoking, body mass index (BMI), waist circumference, pulse, alanine aminotransferase (ALT), alkaline phosphatase (ALP), aspartate aminotransferase (AST), blood urea nitrogen (BUN), creatinine (Cr), globulin, glycohemoglobin, calcium, triglyceride (TG), total cholesterol (TC), LDL-cholesterol (LDL), HDL-cholesterol (HDL), fasting blood glucose (FBG), gamma-glutamyl transferase (γGT), total protein (TP) and serum uric acid (sUA).

We excluded subjects with missing independent or dependent variables. For missing continuous variables, we use the median to fill in. For missing categorical variables, we separate the missing group as a group. All the covariate acquisition processes and any detailed information can be found at www.cdc.gov/nchs/nhanes/.

### Statistical analyses

R statistical programming language (version 3.4.3)^1^ and EmpowerStats software^2^ were applied to perform statistical analysis. A two-sided *p* < 0.05 was considered to be statistically significant. We used the weighted analysis as recommended by the NCHS Analysis Guide to maintain national representation. The continuous variables were characterized by mean ± standard deviation, or as median and interquartile range, as appropriate. The categorical variables were presented as a percentage. The *P*-value was calculated using a weighted chi-squared test for categorical variables and a weighted linear regression model for continuous variables.

The association between vitamin C and SBP or DBP was evaluated by multivariable linear regression analysis. To further analyze the relationship between vitamin C and SBP or DBP, we used the following three models: Model 1: No adjustment for variables; Model 2: Adjusted for sex, age, and race/ethnicity; Model 3: Adjusted for sex, age, race/ethnicity, marital status, education level, ratio of family income to poverty, BMI, smoking states, pulse, ALT, ALP, AST, BUN, globulin, Cr, TG, TC, LDL, HDL, glycohemoglobin, TP, FBG, γGT, and sUA.

For further analyses, we performed subgroup analysis stratified by sex and age subsequently. A weighted generalized additive model and a smooth curve fitting were performed to address non-linearity between vitamin C and SBP or DBP. When non-linearity was discovered, we first calculated the vital inflection point using a recursive algorithm and then performed a weighted two-piecewise linear regression model on both sides of the inflection point.

## Results

Table 1 shows the general description of weighed characteristics of all 2533 subjects enrolled in the study based on the quartiles of vitamin C (Q1:1.87-32.1, Q2: 32.1-51.6, Q3:51.6-68.1, Q4:68.1-191). Of all these participants, the average age was 42.11 ± 15.39 years old,46.76% were males, 53.24% were females, 10.94% were Mexican Americans,61.74% were Non-Hispanic Whites, 9.19% were Non-Hispanic Blacks, and 18.13% were other race/ethnicity. Among the four groups stratified by quartile of vitamin C, ALT, ALP, AST, Cr, globulin, GGT, calcium, TP, UA, BMI, waist circumference, pulse, SBP, DBP, AGE, ratio of family income to poverty, glycohemoglobin, FBG, HDL_C, TG were all of great statistical significance (*p* < 0.05). Moreover, alcohol consumption, smoking states, education levels, marital status, diabetes rate were comparable among the quartile groups.

**TABLE 1:** Description of the participants included in the study.

Quartile of vitamin C	Q1 (1.87-32.1) *N* = 629	Q2 (32.1-51.6) *N* = 637	Q3 (51.6-68.1) *N* = 630	Q4 (68.1-191) *N* = 637	ALL *N* = 2533	*P*-value
Age (years old)	40.76 ± 14.71	40.09 ± 14.15	41.85 ± 14.80	45.43 ± 17.04	42.11 ± 15.39	<0.0001
Sex (%)						<0.0001
Male	57.38	50.31	51.27	28.79	46.76	
Female	42.62	49.69	48.73	71.21	53.24	
Race/Ethnicity (%)						<0.0001
Mexican American	9.06	15.28	10.98	8.99	10.94	
Other race	15.44	22.20	19.95	15.38	18.13	
Non-Hispanic Whites	67.73	48.86	60.16	68.60	61.74	
Non-Hispanic Blacks	7.78	13.65	8.91	7.03	9.19	
ALT (IU/L)	24.09 ± 19.87	22.01 ± 14.96	21.31 ± 12.04	19.68 ± 12.23	21.74 ± 15.15	<0.0001
ALP (IU/L)	80.87 ± 32.70	75.73 ± 23.27	70.26 ± 20.81	70.72 ± 22.46	74.28 ± 25.61	<0.0001
AST (IU/L)	22.11 ± 16.75	20.61 ± 9.11	21.36 ± 8.41	20.58 ± 7.48	21.18 ± 11.09	0.0439
BUN (mmol/L)	4.90 ± 1.57	4.99 ± 1.45	5.00 ± 1.37	5.00 ± 1.59	4.97 ± 1.50	0.5688
Cr (umol/L)	77.10 ± 18.02	76.45 ± 17.54	73.79 ± 16.92	70.87 ± 15.97	74.46 ± 17.29	< 0.0001
Globulin (g/L)	30.16 ± 4.02	30.64 ± 3.99	29.65 ± 3.44	29.09 ± 3.83	29.85 ± 3.86	<0.0001
GGT (IU/L)	34.09 ± 56.40	28.33 ± 30.75	22.06 ± 15.20	19.34 ± 16.38	25.79 ± 34.35	<0.0001
Calcium (mmol/L)	2.31 ± 0.09	2.32 ± 0.08	2.31 ± 0.08	2.32 ± 0.08	2.32 ± 0.08	0.0400
TP (g/L)	70.82 ± 4.07	71.65 ± 3.99	70.96 ± 3.66	70.55 ± 4.22	70.97 ± 4.01	<0.0001
UA (umol/L)	314.61 ± 79.31	320.20 ± 84.90	310.82 ± 78.74	280.60 ± 67.52	306.02 ± 79.11	<0.0001
BMI (kg/m^2^)	30.62 ± 8.32	29.62 ± 6.59	28.45 ± 6.05	26.44 ± 5.62	28.74 ± 6.89	<0.0001
Waist circumference (cm)	102.39 ± 19.19	99.21 ± 14.89	96.61 ± 15.79	91.56 ± 14.74	97.34 ± 16.76	<0.0001
Pulse (bpm)	73.38 ± 12.25	72.58 ± 11.41	70.59 ± 9.80	71.05 ± 11.49	71.86 ± 11.31	<0.0001
SBP (mmHg)	117.35 ± 11.01	117.26 ± 10.71	115.05 ± 11.14	113.79 ± 11.35	115.79 ± 11.17	<0.0001
DBP (mmHg)	53.91 ± 7.43	53.77 ± 7.59	53.01 ± 7.35	50.84 ± 7.55	52.85 ± 7.58	<0.0001
Glycohemoglobin (%)	5.58 ± 0.88	5.57 ± 0.90	5.44 ± 0.67	5.39 ± 0.49	5.49 ± 0.75	<0.0001
FBG (mmol/L)	6.09 ± 1.04	6.12 ± 1.37	5.92 ± 0.79	5.97 ± 0.59	6.02 ± 0.98	0.0004
HDL_C (mmol/L)	1.29 ± 0.33	1.33 ± 0.38	1.42 ± 0.38	1.56 ± 0.40	1.40 ± 0.39	<0.0001
TC (mmol/L)	4.89 ± 1.05	4.94 ± 1.07	4.85 ± 0.96	4.87 ± 1.00	4.88 ± 1.02	0.4838
TG (mmol/L)	1.21 ± 0.55	1.32 ± 1.32	1.15 ± 0.60	1.14 ± 0.39	1.20 ± 0.78	0.0001
LDL_C (mmol/L)	2.86 ± 0.64	2.87 ± 0.67	2.79 ± 0.63	2.81 ± 0.57	2.83 ± 0.63	0.0746
Ratio of family income to poverty	2.81 ± 1.64	2.93 ± 1.52	3.26 ± 1.55	3.16 ± 1.54	3.05 ± 1.57	<0.0001
Alcohol consumption (%)						0.0129
Yes	24.44	18.80	21.92	26.27	22.99	
No	75.56	81.20	78.08	73.73	77.01	
Education levels (%)						<0.0001
Lower than high school	12.81	12.80	8.14	8.05	10.33	
High school	65.60	54.25	54.16	46.73	55.14	
College or above	21.59	32.95	37.70	45.16	34.51	
Missing				0.06	0.01	
Marital status (%)						0.0103
Married/cohabiting/remarried	58.03	59.76	67.92	60.23	61.61	
Unmarried/divorced/widowed	41.94	40.24	32.08	39.71	38.37	
Missing/Refused	0.03			0.06	0.02	
Diabetes (%)						0.0003
Yes	6.84	6.14	2.80	3.05	4.63	
No	93.16	93.86	97.20	96.95	95.37	
Smoking (%)						<0.0001
Yes	55.99	35.82	35.04	30.85	39.41	
No	44.01	64.18	64.96	69.15	60.59	

**FIGURE 2:**
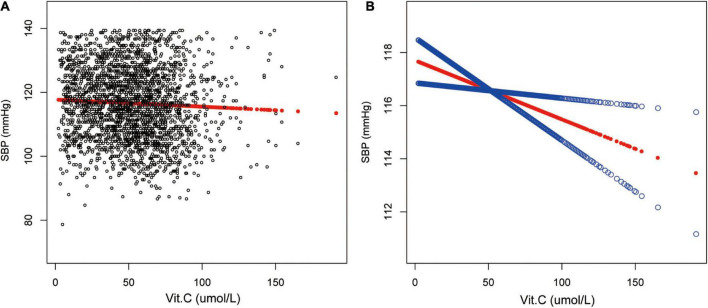
Relationship between vitamin C and SBP. **(A)** Each black point represents a sample. **(B)** The red line represents the smooth curve fit between variables. In comparison, blue bands represent the 95% CI. Sex, age, race/ethnicity, marital status, education level, ratio of family income to poverty, BMI, smoking states, pulse, ALT, ALP, AST, BUN, globulin, Cr, TG, TC, LDL, HDL, glycohemoglobin, TP, FBG, γGT and sUA were adjusted.

Vitamin C and SBP were reversely correlated in the fully adjusted model (β = −0.02, 95% CI: −0.03 to -0.00, *p* = 0.0306). The test for trend among the vitamin C quartile groups was statistically significant (*p* = 0.037). In sub-analysis stratified by age, sex, race/ethnicity, this negative association was observed only in non-Hispanic White [β = −0.03, 95% CI: −0.06 to −0.01, *p* = 0.0088] (Table 2 and Figure 2).

**TABLE 2:** Association between serum vitamin C (umol/L) and SBP (mmHg).

Outcome	Model 1, β (95% CI), *p*	Model 2, β (95% CI), *p*	Model 3, β (95% CI), *p*
SBP	−0.05 (−0.07, −0.03) < 0.0001	−0.05 (−0.06, −0.03) < 0.0001	−0.02 (−0.03, −0.00) 0.0306
**Quartiles of vitamin C**			
Q1 (1.87-32.1)	Reference	Reference	Reference
Q2 (32.1-51.6)	−0.09 (−1.35, 1.16) 0.8829	0.16 (−1.02, 1.35) 0.7876	1.17 (0.01, 2.33) 0.0489
Q3 (51.6-68.1)	−2.30 (−3.50, −1.10) 0.0002	−2.28 (−3.41, −1.15) < 0.0001	−0.74 (−1.87, 0.38) 0.1936
Q4 (68.1-191)	−3.56 (−4.77, −2.36) < 0.0001	−3.14 (−4.30, −1.98) < 0.0001	−1.84 (−2.02, −0.34) 0.0149
P for trend	<0.001	<0.001	0.037
**Stratified by age**			
Age < 50years	−0.10 (−0.12, −0.08) < 0.0001	−0.07 (−0.09, −0.05) < 0.0001	−0.02 (−0.04, 0.00) 0.0788
Age ≥ 50 years	−0.02 (−0.04, 0.01) 0.1592	−0.01 (−0.04, 0.01) 0.4050	−0.00 (−0.03, 0.02) 0.8273
**Stratified by sex**			
Male	−0.03 (−0.05, −0.00) 0.0385	−0.04 (−0.07, −0.01) 0.0022	−0.02 (−0.04, 0.01) 0.2281
Female	−0.03 (−0.06, −0.01) 0.0024	−0.05 (−0.07, −0.03) < 0.0001	−0.02 (−0.04, 0.00) 0.0827
**Stratified by race/ethnicity**			
Mexican American	−0.03 (−0.08, 0.02) 0.3053	−0.02 (−0.06, 0.03) 0.4547	−0.00 (−0.05, 0.05) 0.9753
Other Race/Ethnicity	−0.05 (−0.08, −0.01) 0.0075	−0.02 (−0.05, 0.01) 0.2763	−0.02 (−0.05, 0.02) 0.3313
Non-Hispanic White	−0.05 (−0.08, −0.03) 0.0001	−0.06 (−0.08, −0.03) < 0.0001	−0.03 (−0.06, −0.01) 0.0088
Non-Hispanic Black	−0.07 (−0.12, −0.03) 0.0012	−0.04 (−0.09, −0.00) 0.0473	−0.02 (−0.06, 0.03) 0.4844

Model 1: No adjustment for variables.

Model 2: Adjusted for sex, age, and race/ethnicity.

Model 3: Adjusted for sex, age, race/ethnicity, marital status, education level, ratio of family income to poverty, BMI, smoking states, pulse, ALT, ALP, AST, BUN, globulin, Cr, TG, TC, LDL, HDL, glycohemoglobin, TP, FBG, γGT, and sUA.

**FIGURE 3:**
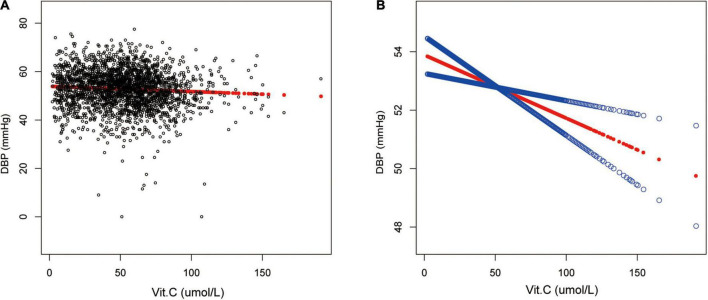
Relationship between vitamin C and DBP. **(A)** Each black point represents a sample. **(B)** The red line represents the smooth curve fit between variables. In comparison, blue bands represent the 95% CI. Sex, age, race/ethnicity, marital status, education level, ratio of family income to poverty, BMI, smoking states, pulse, ALT, ALP, AST, BUN, globulin, Cr, TG, TC, LDL, HDL, glycohemoglobin, TP, FBG, γGT, and sUA were adjusted.

Moreover, a reverse association between vitamin C and DBP in the fully-adjusted model was observed (β = −0.02, 95% CI: −0.04 to −0.01, *p*<0.0011). The trend remained to be of statistical significance among the vitamin C quartile groups as well (*p* < 0.001). Additional sub-analysis stratified by age, sex, race/ethnicity showed this negative relationship existed in both male (β = −0.02,95% CI: −0.04 to −0.00, *p* = 0.0231) and female (β = −0.02,95% CI: −0.04 to −0.04, *p* = 0.0024), and in those older than 50 years old (β = −0.04,95% CI: −0.06 to −0.02, *p*<0.0011) as well as in non-Hispanic White (β = −0.03,95% CI: −0.05 to −0.01, *p* = 0.0020) (Table 3 and Figure 3).

**TABLE 3:** Association between serum vitamin C (umol/L) and DBP (mmHg).

Outcome	Model 1, β (95% CI), *p*	Model 2, β (95% CI), *p*	Model 3, β (95% CI), *p*
DBP	−0.04 (−0.05, −0.03) < 0.0001	−0.03 (−0.04, −0.02) < 0.0001	−0.02 (−0.04, −0.01) < 0.0001
**Quartiles of vitamin C**			
Q1 (1.87-32.1)	Reference	Reference	Reference
Q2 (32.1-51.6)	−0.14 (−0.99, 0.71) 0.7481	0.04 (−0.80, 0.89) 0.9198	0.13 (−0.72, 0.98) 0.7642
Q3 (51.6-68.1)	−0.90 (−1.71, −0.09) 0.0294	−0.81 (−1.61, −0.00) 0.0489	−0.58 (−1.40, 0.24) 0.1663
Q4 (68.1-191)	−3.07 (−3.88, −2.25) < 0.0001	−2.58 (−3.40, −1.75) < 0.0001	−2.08 (−2.94, −1.22)<0.0001
P for trend	<0.001	<0.001	<0.001
**Stratified by age**			
Age < 50years	−0.04 (−0.05, −0.03) 0.0001	−0.03 (−0.04, −0.01) 0.0005	−0.01 (−0.02, 0.01) 0.2574
Age ≥ 50 years	−0.04 (−0.05, −0.02) 0.0001	−0.03 (−0.05, −0.01) 0.0007	−0.04 (−0.06, −0.02) < 0.0001
**Stratified by sex**			
Male	−0.03 (−0.04, −0.01) 0.0055	−0.03 (−0.05, −0.01) 0.0028	−0.02 (−0.04, −0.00) 0.0231
Female	−0.03 (−0.04, −0.02) < 0.0001	−0.03 (−0.05, −0.02) < 0.0001	−0.02 (−0.04, −0.01) 0.0024
**Stratified by race/ethnicity**			
Mexican American	−0.04 (−0.08, −0.01) 0.0221	−0.04 (−0.07, −0.00) 0.0338	−0.02 (−0.06, 0.01) 0.2127
Other Race/Ethnicity	−0.02 (−0.04, 0.00) 0.0529	−0.01 (−0.03, 0.01) 0.2740	−0.02 (−0.04, 0.01) 0.1600
Non-Hispanic White	−0.04 (−0.06, −0.02) < 0.0001	−0.03 (−0.05, −0.01) 0.0007	−0.03 (−0.05, −0.01) 0.0020
Non-Hispanic Black	−0.04 (−0.08, −0.01) 0.0091	−0.03 (−0.06, 0.00) 0.0658	−0.02 (−0.06, 0.01) 0.2060

Model 1: No adjustment for variables.

Model 2: Adjusted for sex, age, and race/ethnicity.

Model 3: Adjusted for sex, age, race/ethnicity, marital status, education level, ratio of family income to poverty, BMI, smoking states, pulse, ALT, ALP, AST, BUN, globulin, Cr, TG, TC, LDL, HDL, glycohemoglobin, TP, FBG, γGT and sUA.

**FIGURE 4:**
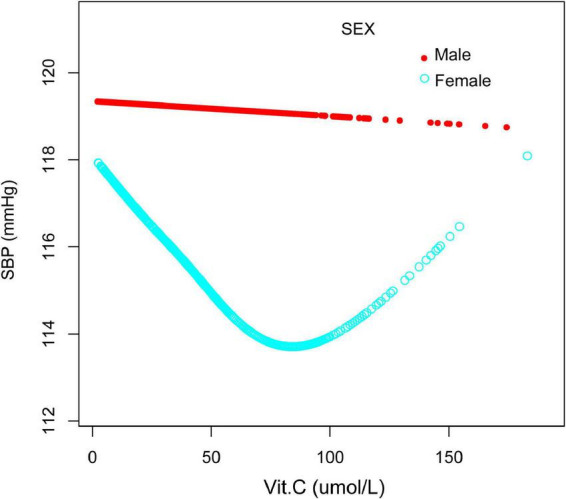
The relationship between vitamin C and SBP, stratified by sex. Age, race/ethnicity, marital status, education level, ratio of family income to poverty, BMI, smoking states, pulse, ALT, ALP, AST, BUN, globulin, Cr, TG, TC, LDL, HDL, glycohemoglobin, TP, FBG, γGT, and sUA were adjusted.

**FIGURE 5:**
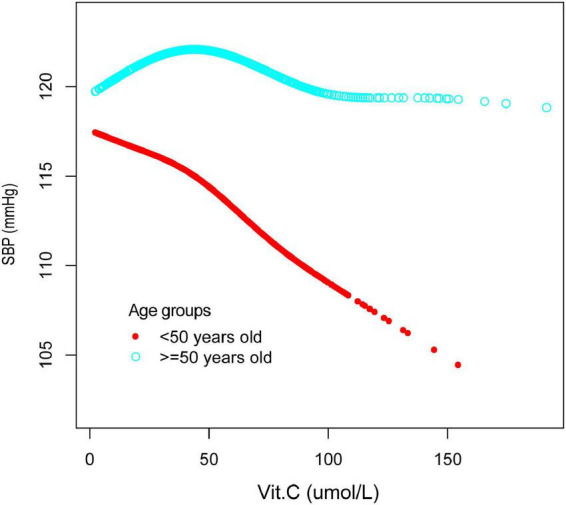
The relationship between vitamin C and SBP, stratified by age. Sex, race/ethnicity, marital status, education level, ratio of family income to poverty, BMI, smoking states, pulse, ALT, ALP, AST, BUN, globulin, Cr, TG, TC, LDL, HDL, glycohemoglobin, TP, FBG, γGT, and sUA were adjusted.

Additionally, we also performed a weighted generalized additive model and a smooth curve fitting stratified by age and sex to detect the non-linear association between vitamin C and SBP as well as DBP and further confirm the results. A U-shaped association and a reverse one were detected between vitamin C and SBP in females and those older than 50years, respectively. We further calculated the inflection points at 90.3μmol/L for females and 40μmol/L for age ≥ 50 years (Table 4 and Figures 4, 5).

**TABLE 4:** Threshold effect analysis of vitamin C and SBP using two-precise linear regression.

SBP	Adjusted β (95% CI), *p*
**Female**	
Fitting by a standard linear model	−0.02 (−0.04, 0.00) 0.0706
**Fitting by two precise linear model**	
Inflection point	90.3
Vitamin C < 90.3 umol/L	−0.03 (−0.06, −0.01) 0.0052
Vitamin C > 90.3 umol/L	0.06 (−0.11, −0.03) 0.0370
Log-likelihood ratio	<0.001
**Age ≥ 50 years**	
Fitting by a standard linear model	−0.01 (−0.04, 0.02) 0.5457
**Fitting by two precise linear model**	
Inflection point	40
Vitamin C < 40 umol/L	0.09 (0.00, 0.18) 0.0407
Vitamin C > 40 umol/L	−0.04 (−0.08, −0.00) 0.0361
Log-likelihood ratio	0.019

Sex, age, race/ethnicity, marital status, education level, ratio of family income to poverty, BMI, smoking states, pulse, ALT, ALP, AST, BUN, globulin, Cr, TG, TC, LDL, HDL, glycohemoglobin, TP, FBG, γGT and sUA were adjusted.

**FIGURE 6:**
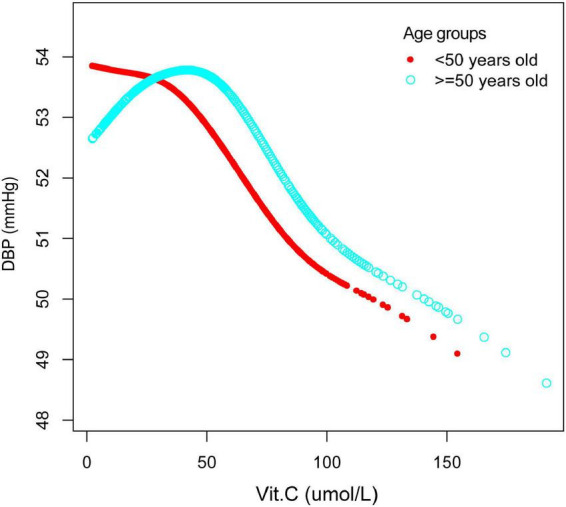
The association between vitamin C and DBP, stratified by sex. Age, race/ethnicity, marital status, education level, ratio of family income to poverty, BMI, smoking states, pulse, ALT, ALP, AST, BUN, globulin, Cr, TG, TC, LDL, HDL, glycohemoglobin, TP, FBG, γGT, and sUA were adjusted.

**FIGURE 7:**
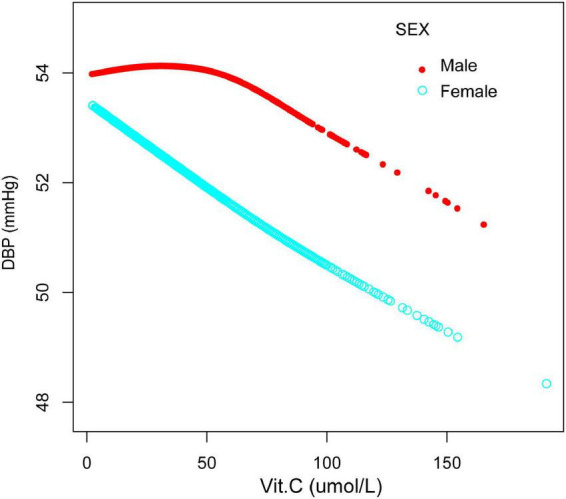
The association between vitamin C and DBP, stratified by age. Sex, race/ethnicity, marital status, education level, ratio of family income to poverty, BMI, smoking states, pulse, ALT, ALP, AST, BUN, globulin, Cr, TG, TC, LDL, HDL, glycohemoglobin, TP, FBG, γGT, and sUA were adjusted.

Furthermore, we have also observed an inverted U-shaped distribution between vitamin C and DBP in males and those older than 50years. The inflection point calculated by a recursive algorithm of vitamin C in these groups was all 40 μmol/L (Table 5 and Figures 6, 7).

**TABLE 5:** Threshold effect analysis of vitamin C and DBP using two-precise linear regression.

DBP	Adjusted β (95% CI), *p*
**Age ≥ 50 years**	
Fitting by a standard linear model	−0.03 (−0.04, −0.01) 0.0071
**Fitting by two precise linear model**	
Inflection point	40
Vitamin C < 40 umol/L	−0.06 (−0.12, −0.01) 0.0268
Vitamin C > 40 umol/L	−0.01 (−0.04, 0.101) 0.0446
Log-likelihood ratio	0.014
**Male**	
Fitting by a standard linear model	0.02 (−0.04, −0.00) 0.0351
**Fitting by two precise linear model**	
Inflection point	40
Vitamin C < 40 umol/L	0.05 (−0.00, 0.10) 0.0509
Vitamin C > 40 umol/L	−0.06 (−0.09, −0.03) 0.0002
Log-likelihood ratio	0.002

Sex, age, race/ethnicity, marital status, education level, ratio of family income to poverty, BMI, smoking states, pulse, ALT, ALP, AST, BUN, globulin, Cr, TG, TC, LDL, HDL, glycohemoglobin, TP, FBG, γGT, and sUA were adjusted.

Finally, according to the smoothing plot, we applied a two-piecewise linear regression model to examine vitamin C’s threshold effect on SBP and DBP (Tables 4, 5).

## Discussion

To our knowledge, this is the first analysis to examine the relationship between vitamin C and blood pressure in NHANES. Several significant findings were uncovered in this cross-sectional study. The most prominent finding to emerge from the present study is that vitamin C was negatively associated with SBP and DBP. Sex, age, race/ethnicity might be major contributors affecting this reverse relationship. By quartile of serum vitamin C, we found that those in the highest quartile had 1.84 and 2.08 mmHg lower systolic and diastolic blood pressure, respectively, than those in the lowest quartile. A U-shaped association between vitamin C and SBP in females and an inverted one between vitamin C and DBP in males were detected, respectively. We further calculated the inflection points at 90.3 μmol/L for females and 40 μmol/L for males. It is somewhat surprising that a reverse U-shaped distribution between vitamin C and SBP and DBP in people over 50 was detected, and the point of inflection of vitamin C were all located at 40 μmol/L.

Hypertension or high blood pressure, a primary cause of disability and mortality and a leading risk factor for cardiovascular diseases globally, is a complex human disorder with diverse etiology (1, 30, 31). Continually improving and standardized management and monitoring of blood pressure are extraordinary to humans. The early detection and treatment of prehypertensive or hypertensive populations has a major role to play in reducing hypertension incidence. It is the first study to demonstrate that serum vitamin C concentration is associated with elevated blood pressure in healthy populations, as well as a non-curve relationship for specific populations, which implies that lifestyle interventions may have implications for reducing hypertension prevalence. Several observational and interventional studies have demonstrated that multiple dietary factors were associated with an increased risk of developing hypertension and might be served as a potential antihypertensive therapeutic target (5, 32–34). A Chinese longitudinal national study indicated that lower or higher calcium intake during adolescence could increase the risk of hypertension in adulthood (34). Besides, a few scholars believe that vitamin C supplementation may significantly reduce the risk of major cardiovascular events and hypertension (18, 23, 35, 36). A meta-analysis also reported that the administration of vitamin C improved the systolic left ventricular function in heart failure patients (37).

Previous studies have confirmed that vitamin C is a common antioxidant with potential tissue protection and antihypertensive effects (15, 18, 23, 38). The blood pressure lowing potential of vitamin C supplementation has been frequently reported recently in various studies (15, 17, 19, 39). In a meta-analysis conducted by Ran et al. (15), the research staff reported that serum vitamin C concentrations in hypertensive subjects were 15.13 μmol/L, much lower than in normotensive individuals.

The current studies on the relationship between vitamin C and blood pressure with contradictory results were mainly carried out in participants including both normotensive and hypertensive subjects or in patients only with hypertension (17, 40, 41). Besides, as is known, antihypertensive medication used by some participants may have impacted on the relationship between serum vitamin C and blood pressure (42). On the other hand, relatively few studies have focused primarily on those with normal blood pressure. Consistent with previous studies, we also detected for the first time that serum vitamin C is reversely correlated with both SBP and DBP in normotensive subjects, which may have significant implications in the general population to reduce hypertension prevalence in the early stage by external interventions such as vitamin C supplementation.

Several mechanisms by which vitamin C may exert blood pressure lowing effect may be by downregulating antioxidative stress response and anti-inflammatory cytokines, as confirmed in a number of studies (20–22). Moreover, vitamin C may possess cardioprotective effects by ameliorating the cardiac autonomic nerve imbalance and restoring vagal and sympathetic tone to normal range. In an experimental study conducted by Fabiyi-Edebor TD (43), researchers indicated that vitamin C administration restored normal-tension in diabetic rats via ameliorating cardiac autonomic neuropathy. Furthermore, an animal study confirmed that dietary supplementation of vitamin C might play a hypotensive effect by enriching the diversity of gut microbes and reshaping their functions. To our knowledge, gut microbes and their metabolites perform important functions in cardiovascular diseases such as hypertension, myocardial fibrosis, arrhythmia, and atherosclerosis via various routes (44–46). Vitamin C as an antioxidant may counteract the effects of trimethylamine N-oxide, a metabolite derived from intestinal microbes, and exert a pharmacological effect of antihypertensive. In addition, studies have indicated that endothelial dysfunction was an underlying cause of hypertension, and vitamin C supplements restored endothelial function and corrected vascular NO deficiency that could account for its antihypertensive effect (47, 48). Alternatively, vitamin C has been reported to act as a vasodilator, possibly by increasing nitric oxide bioavailability, thereby affecting blood pressure (49). Vitamin C appears to be associated with a reduction in vascular sensitivity to noradrenaline and an increase in endothelium-dependent relaxation due to increased nitric oxide bioavailability, according to a study (50).

The present analyses indicate that the association between vitamin C and blood pressure remains controversial (51), and we speculate that the reason for this paradoxical phenomenon may be related to population differences. Therefore, we performed a stratified analysis and found that different concentrations of vitamin C had different effects on blood pressure in different populations, which may shed light on the current conflicting results. We found an inverted U-shape association between vitamin C and blood pressure for people over 50 years old. Specifically, it is better to maintain the concentrations of vitamin C above 40 μmol/L, for higher levels of vitamin C are associated with lower blood pressure. Lower serum vitamin C concentrations (less than 40 μmol/L) are related to increased blood pressure, which may be related to weak antioxidant effects, but studies are needed to confirm further. Another significant finding is that for females, vitamin C has a U-shaped relationship with SBP, while for males, maintaining serum vitamin C concentrations higher than 40 μmol/L is related to lower DBP. It is the first time revealing a gender difference in the relationship between vitamin C and blood pressure. The reason for this gender difference, according to our speculation, may be related to the interaction between a variety of sex hormones and maybe also associated with an insufficient research sample size. However, more prospective research is needed in the future to explore the underlying causes.

We thought that different serum vitamin C levels should be controlled or intervened to better manage blood pressure in different populations. Unfortunately, there are no studies or guidelines that discuss the optimal level of vitamin C in humans. The results of the present study will serve as a reference for future clinical management of blood pressure in various populations.

In short, our research has important clinical implications for hypertension management, especially for early interventions for those at high risk of developing hypertension. Nevertheless, there are still some shortcomings of the present study: Firstly, since this was a cross-sectional study, the individuals were not followed up, and the relationship between vitamin C and adverse outcomes and causality could not be effectively evaluated. Furthermore, this study did not exclude patients with other diseases that may interfere with blood pressure. Scholars still need to be cautious when facing the results of the study. Finally, additional potential confounding factors, such as dietary factors or physical activities were not taken into consideration.

## Conclusion

Vitamin C was reversely correlated with both SBP and DBP in this cross-sectional analysis. However, a U-shaped relationship and an inverted one were also observed in certain people, which implied that, though vitamin C is considered a vital antioxidant, maintaining vitamin C at appropriate levels may be beneficial according to different populations.

## Data availability statement

The raw data supporting the conclusions of this article will be made available by the authors, without undue reservation.

## Ethics statement

This study was granted ethical approval by the National Center for Health Statistics (NCHS). This study was carried out following the ethical standards of the responsible committee on human experimentation and with the 1975 Helsinki Declaration and its later amendments. Furthermore, written informed consent was received from each subject.

## Author contributions

RH, LS, and JZ contributed to the design, data analysis and interpretation, and drafting of the manuscript. YL and TL contributed to the data interpretation and critically revised the manuscript. RH and TL contributed to the conception, design, data acquisition, analysis and interpretation, and critical review of the manuscript. TL was the article’s guarantor. All authors read and approved the final version of the manuscript.

## Footnotes

1
http://www.Rproject.org

2
http://www.empowerstats.com

## Abbreviations

SBP: systolic blood pressure
DBP: diastolic blood pressure
NHANES: National Health and Nutrition Examination Survey
NCHS: National Center for Health Statistics
MEC: Mobile Inspection Center
BMI: body mass index
MIL: maximum inflation level
AST: aspartate aminotransferase
ALT: alanine aminotransferase
ALP: alkaline phosphatase
BUN: blood urea nitrogen
TP: total protein
FBG: fasting blood glucose
HDL-C: high-density lipoprotein cholesterol
Cr: creatinine
TG: triglyceride
TC: total cholesterol
LDL_C: LDL-cholesterol
γ GT: gamma-glutamyl transferase
sUA: serum uric acid.
