# Supplementary material for: Differential influences of serum vitamin C on blood pressure based on age and sex in normotensive individuals
Source: Front Nutr. 2022 Nov 29;9:986808. doi: 10.3389/fnut.2022.986808 (PMC9745039; doi:10.3389/fnut.2022.986808)
Supplement: Supplementary file 1 [file Table_1.docx]

Description of Laboratory Methodology

Vitamin C (ascorbic acid) in serum is measured using isocratic ultra‐high performance liquid chromatography (UPLC) with electrochemical detection at 450 mV (range 200 nA). One-part serum is mixed with four parts 6% metaphosphoric acid (MPA) to acidify the serum and stabilize ascorbate. The specimen is frozen at ‐70 °C until analysis. After the specimen is thawed at room temperature and centrifuged at 3,000 rpm, the supernatant is decanted. This supernatant is mixed with a solution containing trisodium phosphate and dithiothreitol (to reduce dehydroascorbate to ascorbate) and an internal standard (1‐methyl uric acid) to reduce dehydroascorbate to ascorbate. It is re‐acidified with 40% MPA to stabilize the ascorbate. The sample is filtered to remove insoluble material. A 4 μL aliquot is injected onto a C‐18 reversed‐phase column and eluted with a mobile phase containing 14.1 g/L monochloroacetic acid, 0.76 g/L disodium ethylenediamine tetraacetate, 1% (by volume) 10 N sodium hydroxide, and 1.5% (by volume) methanol, adjusted to pH 3.00 ± 0.03 with 10 N sodium hydroxide.  Quantitation is accomplished by comparing the peak area of vitamin C in the unknown with the peak area of a known amount in a calibrator solution. Calculations are corrected based on the peak area of the internal standard in the unknown compared with the peak area of the internal standard in the calibrator solution.

Laboratory Quality Assurance and Monitoring

Serum specimens were processed, stored, and shipped to the Division of Laboratory Sciences, National Center for Environmental Health, Centers for Disease Control and Prevention, Atlanta, GA for analysis.
